# Supplementary material for: Does a school-based intervention increase girls’ sexual and reproductive health attitudes and intentions? Results from a mixed-methods cluster-randomized trial in Burkina Faso
Source: PLOS Glob Public Health. 2023 Dec 11;3(12):e0000910. doi: 10.1371/journal.pgph.0000910 (PMC10712850; doi:10.1371/journal.pgph.0000910)

**S1 Text**

**Table A. Comparison of Those Who Dropped Out with Those Who Stayed in Between Baseline and Endline on Key Baseline Variables**

|  |  | **Dropped out**  **N (%)** | **Stayed in**  **N (%)** |
| --- | --- | --- | --- |
| Arm | Intervention | 154 (51.3%) | 1,018 (49.1%) |
|  | Control | 146 (48.7%) | 1,054 (50.9%) |
| City *** | Ouaga | 89 (29.7%) | 1,083 (52.3%) |
|  | Bobo | 211 (70.3%) | 989 (47.7%) |
| Grade | 4ème | 144 (48.0%) | 948 (45.8%) |
|  | 3ème | 156 (52.0%) | 1,124 (54.3%) |
| School type ** | Public | 56 (18.7%) | 544 (26.3%) |
|  | Private | 244 (81.3%) | 1,528 (73.8%) |
| Age | 14 | 58 (19.3%) | 39 (19.1%) |
|  | 15 | 74 (24.7%) | 486 (23.5%) |
|  | 16 | 73 (24.3%) | 471 (22.7%) |
|  | 17 | 54 (18.0%) | 375 (18.1%) |
|  | 18 | 41 (13.7%) | 345 (16.7%) |
| Wealth | Lowest | 70 (23.3%) | 442 (21.3%) |
|  | Middle-low | 71 (23.7%) | 439 (21.2%) |
|  | Middle | 44 (14.7%) | 411 (19.8%) |
|  | Middle-high | 59 (19.7%) | 392 (18.9%) |
|  | Highest | 56 (18.7%) | 388 (18.7%) |
| Level of parental education | None/illiterate | 56 (18.7%) | 431 (20.8%) |
|  | Quranic school | 31 (10.3%) | 176 (8.5%) |
|  | Can read/write | 48 (16.0%) | 26 (12.7%) |
|  | Primary and/or partial secondary | 100 (33.3%) | 672 (32.4%) |
|  | Secondary and beyond | 45 (15.0%) | 340 (16.4%) |
|  | Don’t know | 20 (6.7%) | 190 (9.2%) |
| Religion | Muslim | 213 (71.0%) | 1,393 (67.2%) |
|  | Christian | 87 (29.0%) | 679 (32.8%) |
| Intention to use contraception in next 3 months (primary outcome) | Has an intention | 34 (11.3%) | 230 (11.1%) |
|  | No intention | 266 (88.7%) | 1,842 (88.9%) |
| Contraceptive use | Current use | 9 (3.0%) | 81 (3.9%) |
|  | Not currently using | 291 (97.0%) | 1,991 (96.1%) |

*Statistically significant at * p<0.05, **p<0.01, ***p<0.001*

*Note that Christian category also included three girls who reported “other”*

**Table B. Predictors of Intention to Use Contraception in the Next Three Months (N=2,072)**

| **Variables (p value, Wald test)** | **N** | **%** | **OR/95% CI** |
| --- | --- | --- | --- |
| **City (p=0.059)** |  |  |  |
| Ouaga (Reference) | 1083 | 52.3 | 1.00 |
| Bobo | 989 | 47.7 | 0.57 (0.32-1.02) |
| **Type of school (p=0.864)** |  |  |  |
| Private (Reference) | 1528 | 73.7 | 1.00 |
| Public | 544 | 26.3 | 0.95 (0.51-1.76) |
| **Age (year) (p<0.001)** |  |  |  |
| 14 (Reference) | 92 | 4.4 | 1.00 |
| 15 | 426 | 20.6 | 1.43 (0.48-4.27) |
| 16 | 514 | 24.8 | **3.11 (1.08-8.95)** |
| 17 | 437 | 21.1 | **4.27 (1.51-12.10)** |
| 18 | 424 | 20.5 | **6.17 (2.20-17.31)** |
| 19 | 179 | 8.6 | **8.55 (2.71-26.98)** |
| **Grade (p=0.093)** |  |  |  |
| 4ème (Reference) | 948 | 45.8 | 1.00 |
| 3ème | 1124 | 54.2 | 1.25 (0.96-1.61) |
| **Parental education (p=0.342)** |  |  |  |
| None/illiterate (Reference) | 757 | 36.5 | 1.00 |
| Quoranic school | 90 | 4.3 | 0.60 (0.34-1.04) |
| Can read and write | 142 | 6.9 | 1.05 (0.62-1.77) |
| Primary school | 438 | 21.1 | 0.95 (0.66-1.35) |
| Secondary school | 509 | 24.6 | 0.94 (0.65-1.35) |
| Higher than secondary | 71 | 3.4 | 1.09 (0.37-3.19) |
| Don't know | 65 | 3.1 | 0.43 (0.14-1.30) |
| **Religion (p=0.205)** |  |  |  |
| Muslim | 1393 | 67.2 | 1.00 |
| Christian | 679 | 32.8 | 1.18 (0.91-1.52) |
| **Ethnicity (p=0.542)** |  |  |  |
| Mossi | 1228 | 59.3 | 1.00 |
| Other | 844 | 40.7 | 1.08 (0.85-1.38) |
| **Wealth index (p=0.195)** |  |  |  |
| First quintile (Less wealthy) | 442 | 21.3 | 1.00 |
| Second quintile | 380 | 18.3 | 0.71 (0.50-1.01) |
| Third quintile | 447 | 21.6 | 0.81 (0.56-1.16) |
| Fourth quintile | 388 | 18.7 | 0.69 (0.45-1.06) |
| Fifth quintile (Most wealthy) | 415 | 20.0 | **0.65 (0.43-0.97)** |
| **Have you ever had sexual intercourse? (p<0.001)** |  |  |  |
| Has never had sex (Reference) | 1681 | 81.1 | 1.00 |
| Yes | 391 | 18.9 | **11.18 (7.52-16.62)** |
| **Are you currently doing anything to avoid a pregnancy? (p<0.001)** |  |  |  |
| No, don’t know, and prefer not to answer | 1841 | 88.9 | 1.00 |
| Yes | 231 | 11.1 | **18.93 (12.02-29.79)** |
| **How much of an impact has COVID-19 had on you overall? (p=0.106)** |  |  |  |
| Not at all (Reference) | 227 | 11.0 | 1.00 |
| Somewhat | 271 | 13.1 | **1.83 (1.09-3.07)** |
| A little | 344 | 16.6 | 1.13 (0.64-1.98) |
| A lot | 1230 | 59.4 | 1.33 (0.84-2.11) |
| **How much of an impact has COVID-19 had on you overall? (p=0.178)** |  |  |  |
| Not at all (Reference) | 227 | 11.0 | 1.00 |
| Somewhat, a little, or a lot | 1845 | 89.0 | 1.35 (0.87-2.10) |
| **How much anxiety have you felt on a daily basis because of COVID-19? (p=0.283)** |  |  |  |
| Not at all (Reference) | 121 | 5.8 | 1.00 |
| Somewhat | 218 | 10.5 | 1.51 (0.78-2.93) |
| A little | 515 | 24.9 | 0.94 (0.52-1.68) |
| A lot | 1218 | 58.8 | 1.22 (0.69-2.14) |
| **How much anxiety have you felt on a daily basis because of COVID-19? (p=0.554)** |  |  |  |
| Not at all | 121 | 5.8 | 1.00 |
| Somewhat, a little, or a lot | 1951 | 94.2 | 1.18 (0.68-2.03) |
| **How often could you leave home to see friends/others during COVID-19? (p=0.177)** |  |  |  |
| Not at all (Reference) | 705 | 34.0 | 1.00 |
| Somewhat | 544 | 26.3 | 1.62 (0.99-2.66) |
| A little | 564 | 27.2 | **1.60 (1.03-2.48)** |
| A lot | 259 | 12.5 | 1.64 (0.86-3.12) |
| **How often could you leave home to see friends/others during** **COVID-19? (p=0.028)** |  |  |  |
| Not at all | 705 | 34.0 | 1.00 |
| Somewhat, a little, or a lot | 1367 | 66.0 | **1.62 (1.05-2.48)** |
| **Health care workers do not like to give advice to young unmarried girls about FP. (p=0.375)** |  |  |  |
| Disagree | 1393 | 67.2 | 1.00 |
| Agree | 657 | 31.7 | 0.82 (0.53-1.27) |
| Prefer not to answer | 22 | 1.1 | -- |
| **I feel confident in my ability to get a contraceptive method, if I wanted to avoid pregnancy. (p<0.001)** |  |  |  |
| Disagree | 501 | 24.2 | 1.00 |
| Agree | 1567 | 75.6 | **4.78 (2.59-8.82)** |
| Prefer not to answer | 4 | 0.2 | -- |
| **I feel confident in my ability to use a contraceptive method, if I wanted to avoid pregnancy. (p<0.001)** |  |  |  |
| Disagree | 560 | 27.0 | 1.00 |
| Agree | 1510 | 72.9 | **4.45 (2.75-7.19)** |
| Prefer not to answer | 2 | 0.1 | -- |
| **I feel confident in my ability to use and get a contraceptive method, if I wanted to avoid pregnancy. (p<0.001)** |  |  |  |
| Disagree | 643 | 31.0 | 1.00 |
| Agree | 1423 | 68.7 | **4.13 (2.54-6.71)** |
| Prefer not to answer | 6 | 0.3 | -- |
| **Modern contraception can be used by girls. (p<0.001)** |  |  |  |
| Disagree | 722 | 34.8 | 1.00 |
| Agree | 1342 | 64.8 | **2.54 (1.82-3.54)** |
| Prefer not to answer | 8 | 0.4 | -- |
| **I feel confident I could use contraception secretly. (p<0.001)** |  |  |  |
| Disagree | 867 | 41.8 | 1.00 |
| Agree | 1195 | 57.7 | **2.10 (1.54-2.85)** |
| Prefer not to answer | 10 | 0.5 | -- |
| **All modern contraceptives have negative side effects that make me not want to use them. (p=0.049)** |  |  |  |
| Disagree | 588 | 28.4 | 1.00 |
| Agree | 1445 | 69.7 | **0.65 (0.42-1.00)** |
| Prefer not to answer | 39 | 1.9 | -- |
| **Modern contraception causes infertility. (p=0.038)** |  |  |  |
| Disagree | 384 | 18.5 | 1.00 |
| Agree | 1671 | 80.6 | **0.65 (0.43-0.98)** |
| Prefer not to answer | 17 | 0.8 |  |
| **If I am having sex and want to avoid pregnancy, modern contraception is the best option. (p<0.001)** |  |  |  |
| Disagree | 405 | 19.5 | 1.00 |
| Agree | 1657 | 80.0 | **2.12 (1.51-2.97)** |
| Prefer not to answer | 10 | 0.5 | -- |

All models are adjusted for clustering of girls with robust standard errors.

NA: Not applicable (Question not asked)

**Table C. Impact of (re)solve on Intention to Use Contraception in the Next Three Months in stratified analyses.**

|  |  | **Unadjusted model** | **Adjusted model 1** | **Adjusted model 2** | **Adjusted model 3** |
| --- | --- | --- | --- | --- | --- |
|  | **n/N (%**^a^**)** | **OR/95% CI** | **OR/95% CI** | **OR/95% CI** | **OR/95% CI** |
| **Private Schools * (N=1,528)** | | | | | |
| Control | 76/785 (6.9%) | 1.00 | 1.00 | 1.00 | 1.00 |
| Intervention | 122/743 (13.4%) | **1.79 (1.07-3.00)** | **1.88 (1.08-3.27)** | **2.13 (1.36-3.35)** | **2.43 (1.62-3.63)** |
| **Public Schools ** (N=544)** | | | | | |
| Control | 46/269 (15.3%) | 1.00 | 1.00 | 1.00 | 1.00 |
| Intervention | 22/253 (7.8%) | **0.41 (0.20-0.83)** | **0.38 (0.16-0.87)** | 0.48 (0.20-1.16) | 0.49 (0.20-1.17) |
| **3ème Grade (N=1,124)** | | | | | |
| Control | 67/543 (9.9%) | 1.00 | 1.00 | 1.00 | 1.00 |
| Intervention | 90/581 (12.7%) | 1.34 (0.77-2.31) | 1.41 (0.77-2.58) | 1.58 (0.97-2.55) | **1.61 (1.01-2.57)** |
| **4ème Grade (N=948)** | | | | | |
| Control | 55/511 (10.3%) | 1.00 | 1.00 | 1.00 | 1.00 |
| Intervention | 54/437 (13.6%) | 1.21 (0.65-2.24) | 1.25 (0.64-2.45) | 1.77 (0.89-3.51) | 1.85 (0.90-3.78) |

*^a^ Cluster-level summaries of the geometric means.*

*All models are adjusted for intention to use contraception at baseline and clustering of girls with robust standard errors.*

*Model 1 adjusted for age. Model 2 adjusted for age, COVID-19 effect on mobility and currently doing something to avoid a pregnancy. Model 3 adjusted for age, COVID-19 effect on mobility, currently doing something to avoid a pregnancy, city, grade, and wealth quintile. Wald test for all models (unadjusted and adjusted) had a p value <0.05.*

* Twelve clusters per arm.

** Four clusters per arm.

**Fig A. Interaction Between Intervention and Ever Having Been Sexually Active on Intention to Use Contraception in the Next Three Months at Endline (N=2,072)**


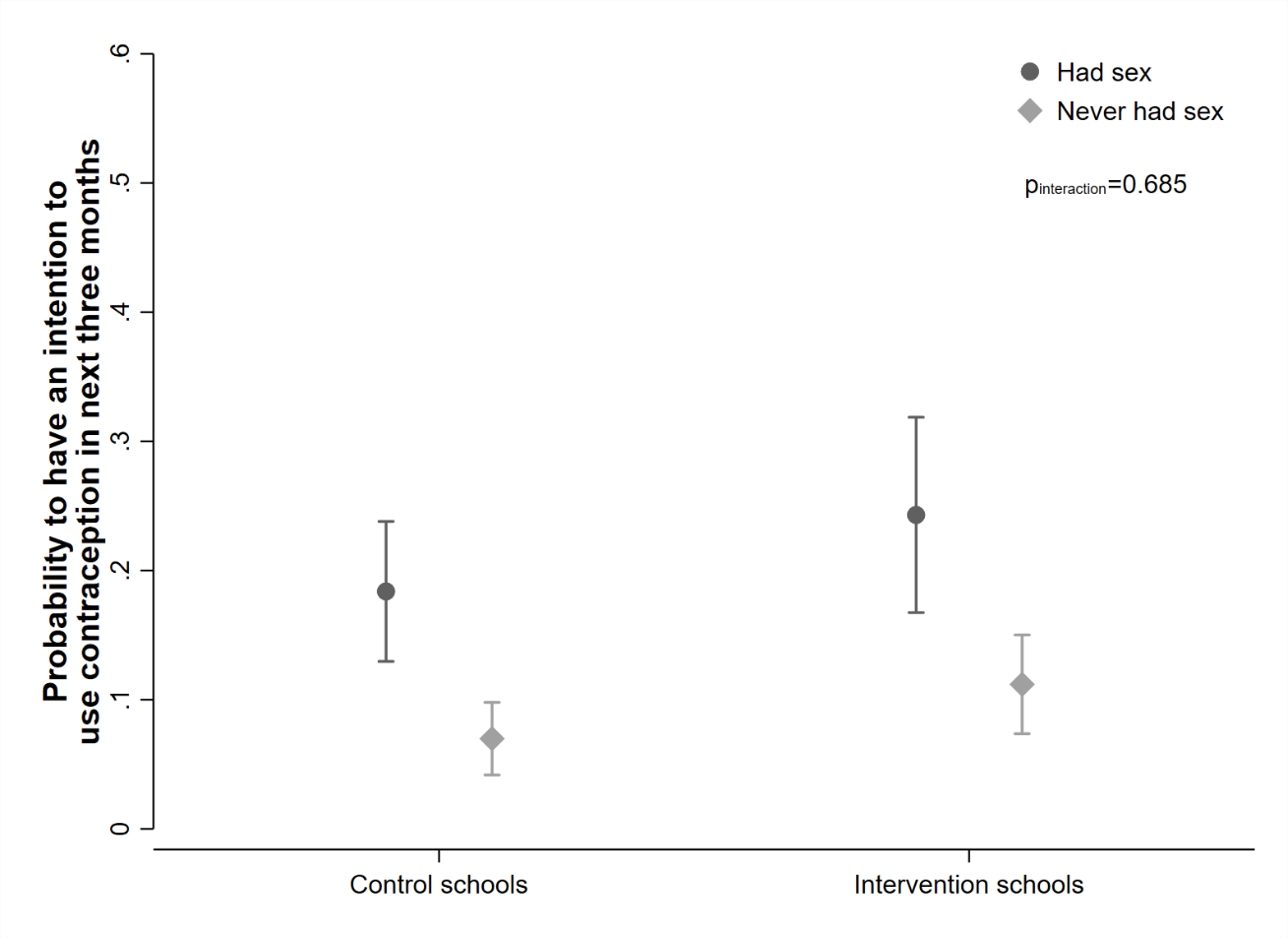


**Fig B. Interaction Between Intervention and Reported Confidence in Ability to Get and Use Contraceptive on Intention to Use Contraception in the Next Three Months at Endline (N=2,066)**


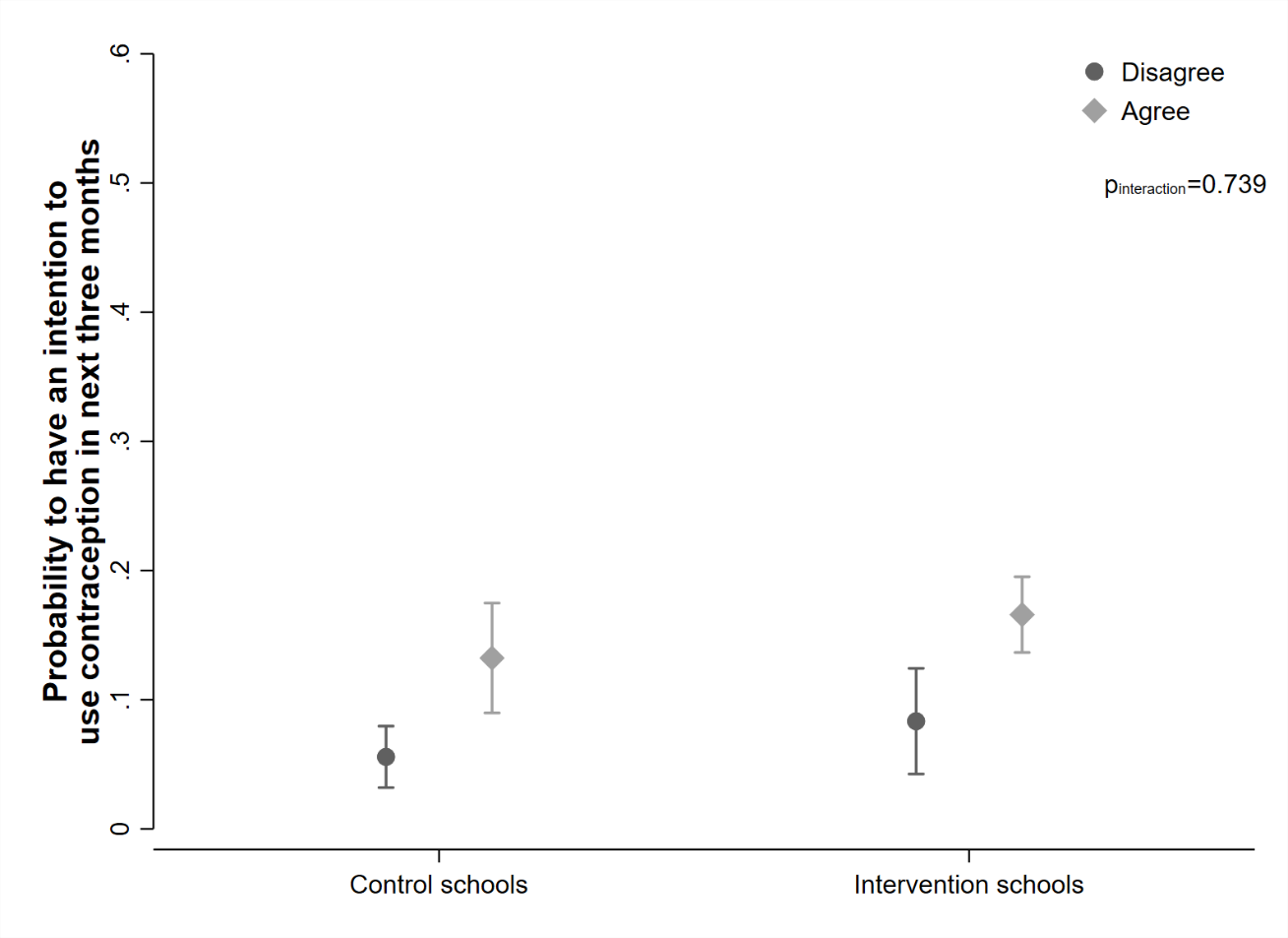


**Fig C. Interaction between Intervention and Responding that Modern Contraception Can be Used by Girls on Intention to Use Contraception in the Next Three Months at Endline (N=2,064)**


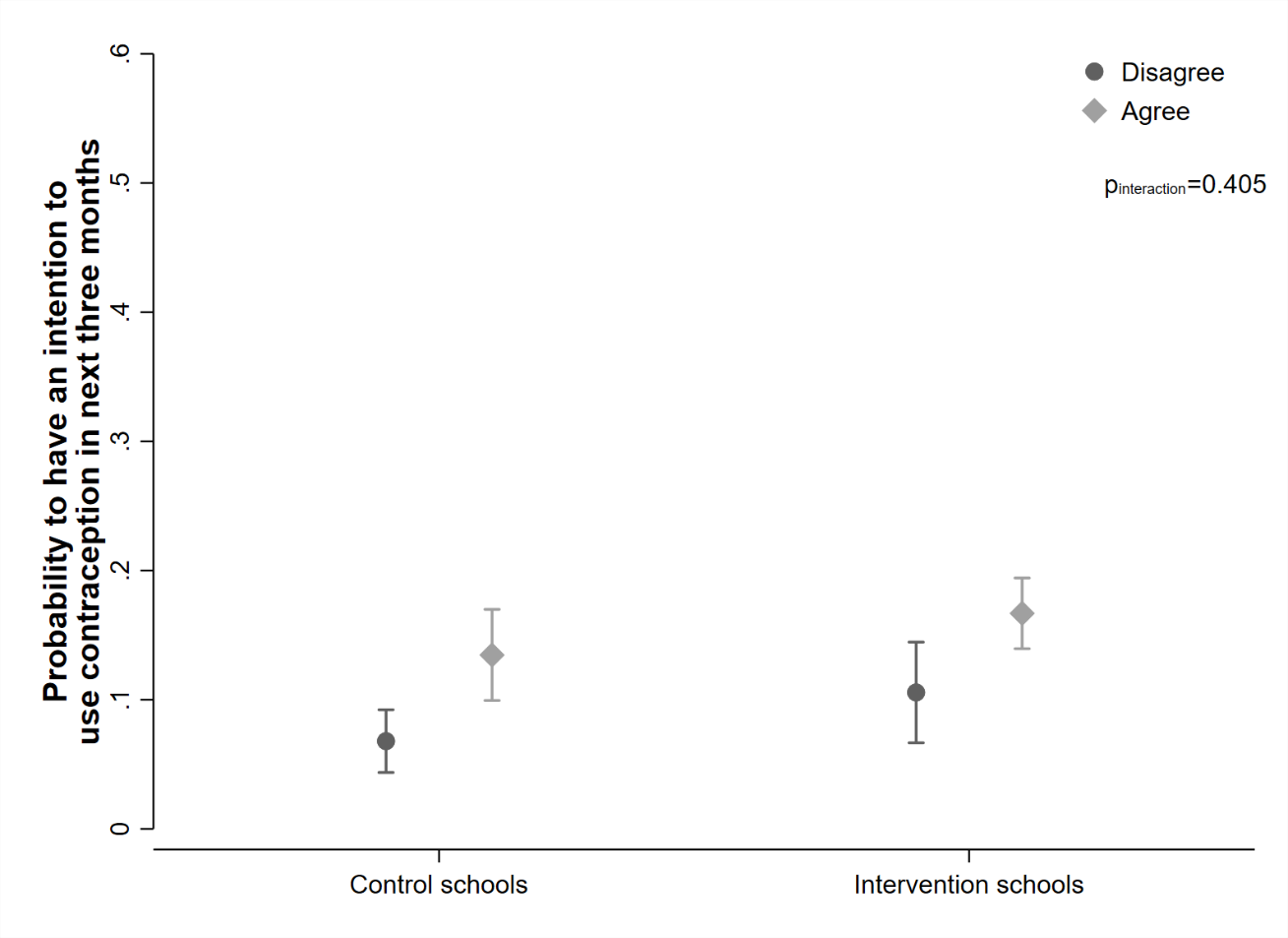


**Fig D. Interaction Between Intervention and Feeling Confident I Could Use Contraception Secretly on Intention to Use Contraception in the Next Three Months at Endline (N=2,062)**


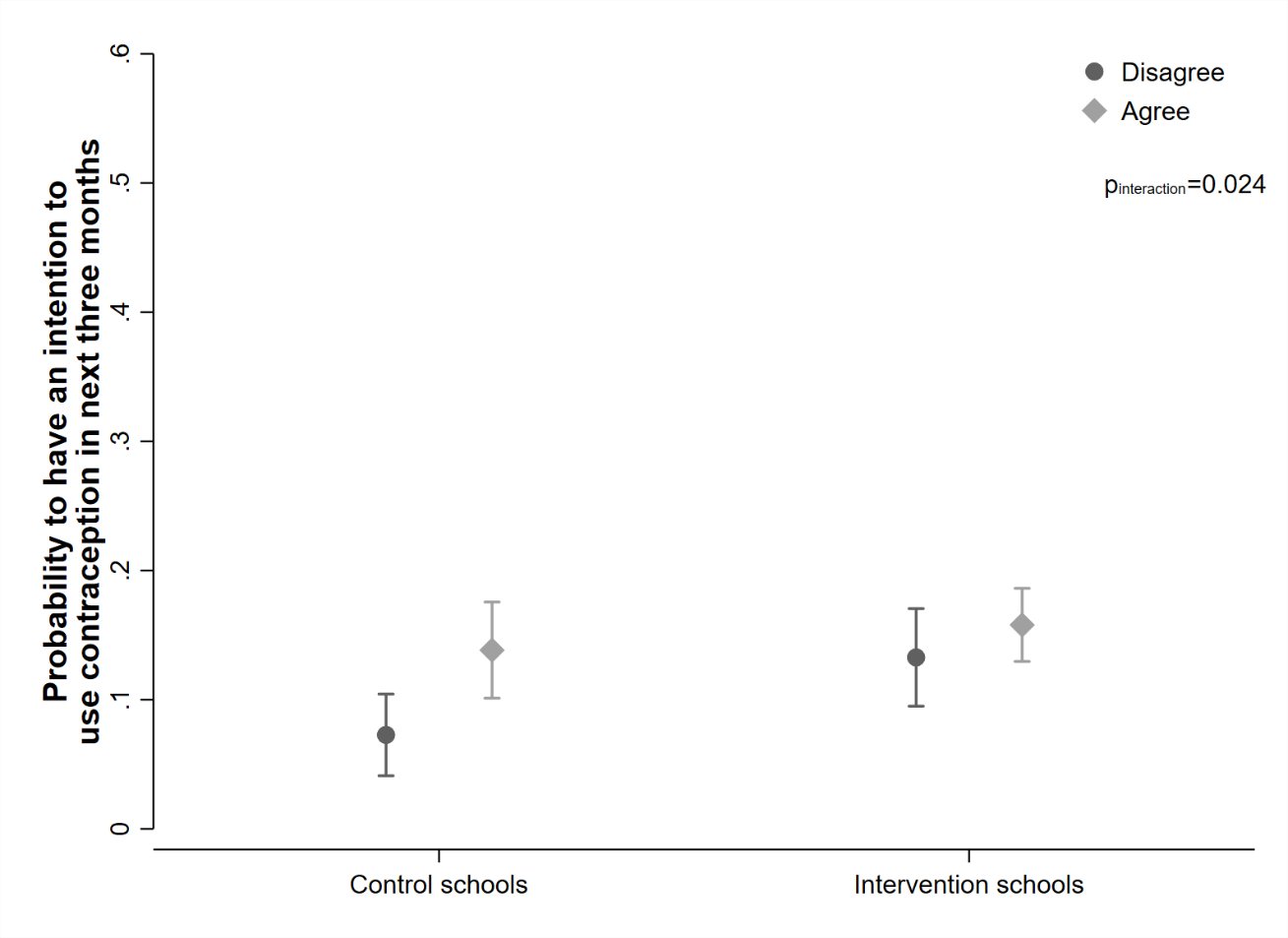


**Fig E. Interaction Between Intervention and Feeling That All Modern Contraceptives Have Negative Side Effects That Make Me Not Want to Use Them on Intention to Use Contraception in the Next Three Months at Endline (N=2,033)**


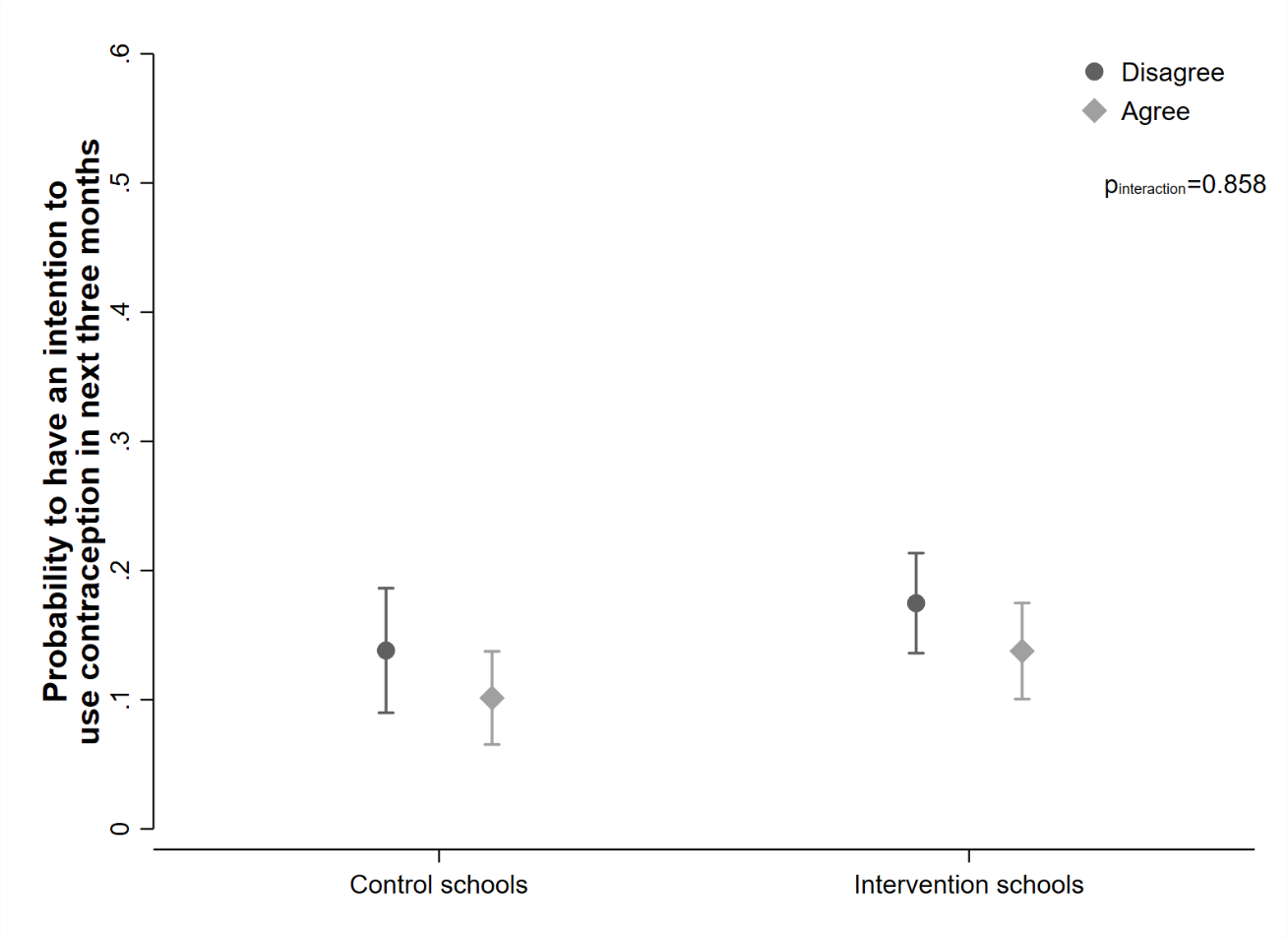


**Fig F. Interaction Between Intervention and Feeling That Modern Contraception Causes Infertility on Intention to Use Contraception in the Next Three Months at Endline (N=2,055)**


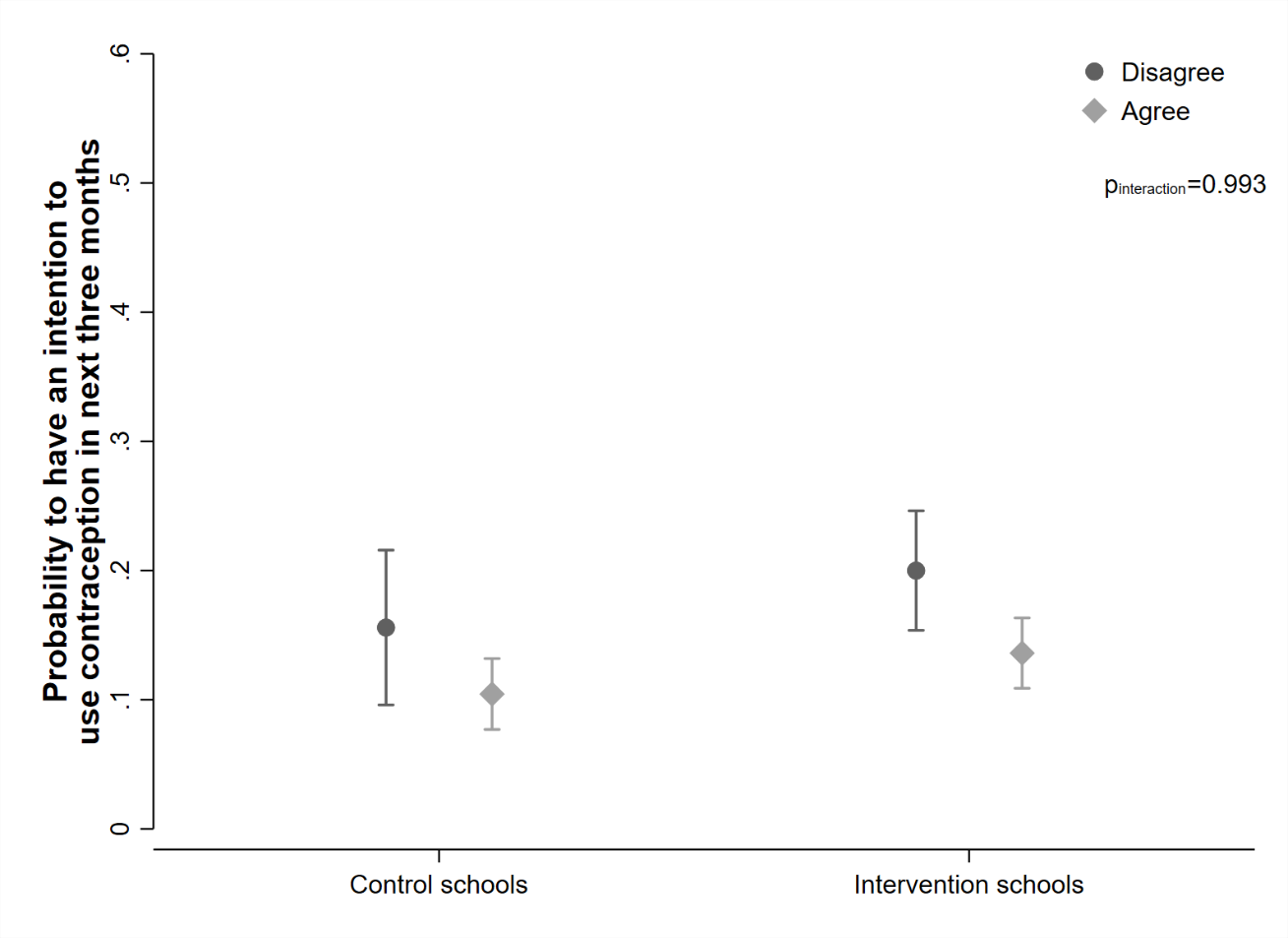


**Fig G. Interaction Between Intervention and Feeling That if I am Having Sex and Want to Avoid Pregnancy Modern Contraception Is Best Option on Intention to Use Contraception in the Next Three Months at Endline (N=2,062)**


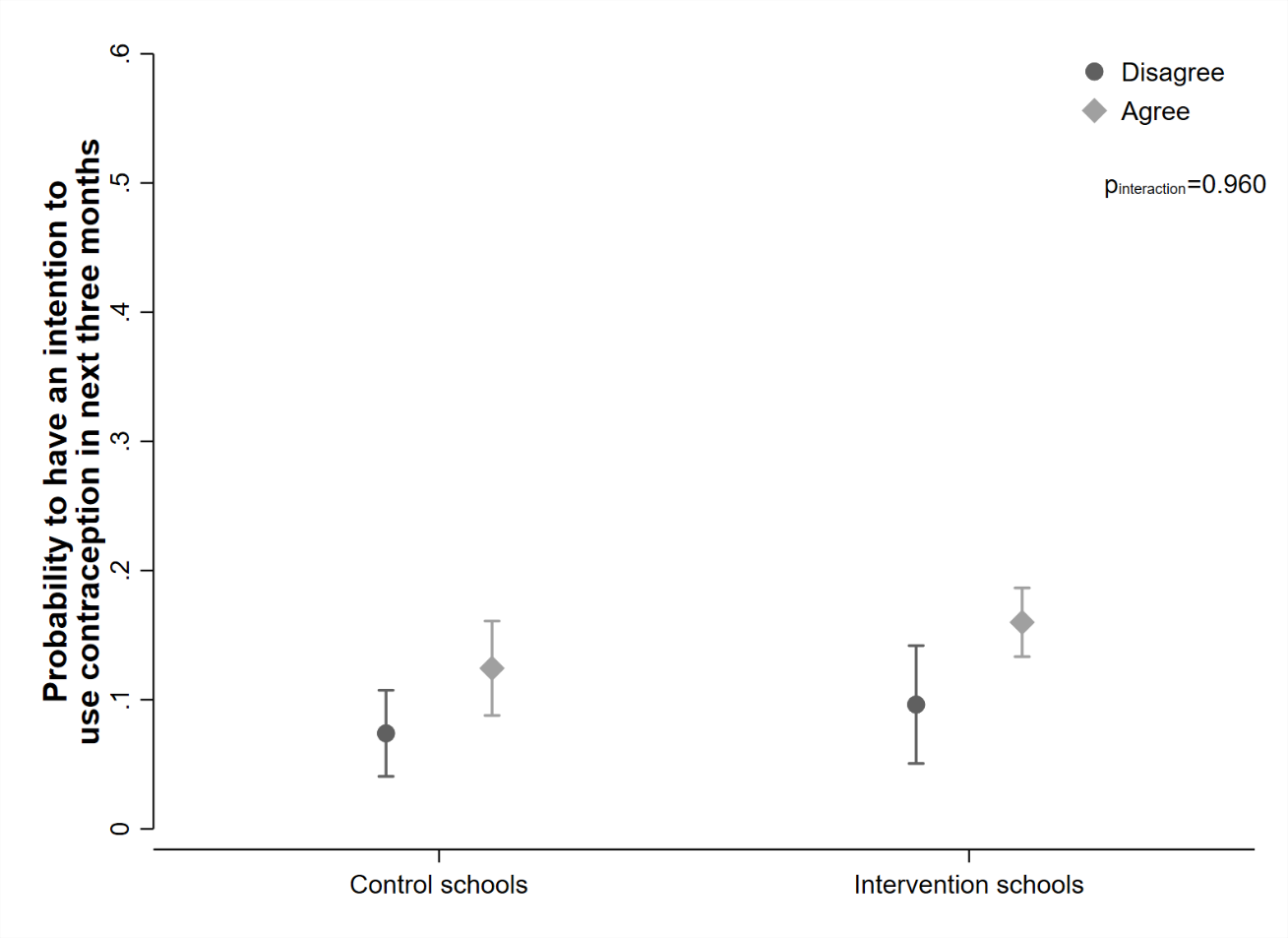


**Fig H. Interaction Between Intervention and Grade on Intention to Use Contraception in the Next Three Months at Endline (N=2,072)**


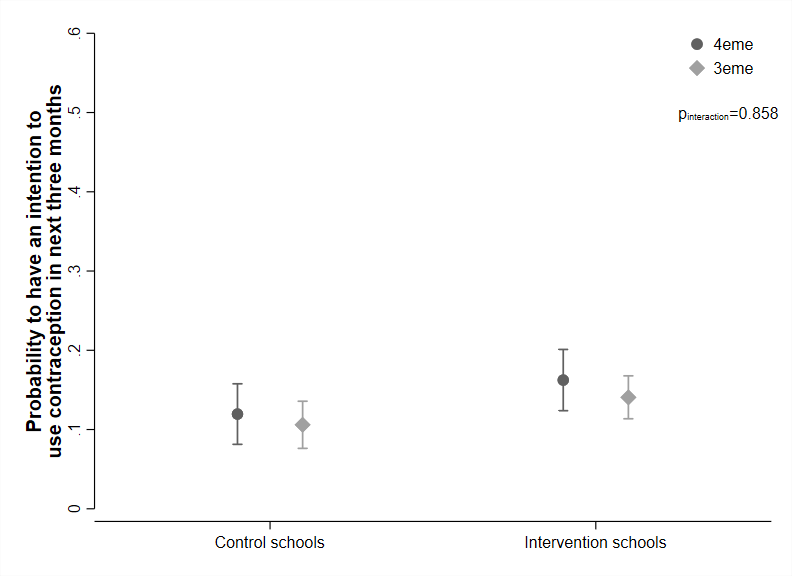


**Fig I. Interaction Between Intervention and School Type on Intention to Use Contraception in the Next Three Months at Endline (N=2,072)**


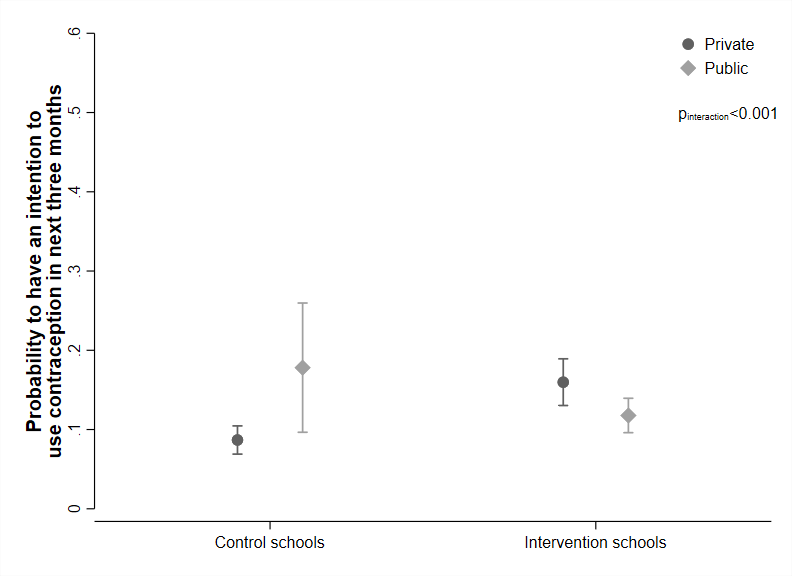

Supplement: S1 Text — (DOCX) [file pgph.0000910.s002.docx]
