# Supplementary material for: Does a school-based intervention increase girls’ sexual and reproductive health attitudes and intentions? Results from a mixed-methods cluster-randomized trial in Burkina Faso
Source: PLOS Glob Public Health. 2023 Dec 11;3(12):e0000910. doi: 10.1371/journal.pgph.0000910 (PMC10712850; doi:10.1371/journal.pgph.0000910)
Supplement: Attachment — Submitted filename: Final Reviewer Response.docx [file pgph.0000910.s004.docx]

**Journal requirements**

1. Please amend your detailed Financial Disclosure statement. This is published with the article. It must therefore be completed in full sentences and contain the exact wording you wish to be published.

a. State the initials, alongside each funding source, of each author to receive each grant.

If you did not receive any funding for this study, please simply state: “The authors received no specific funding for this work.”

**Response**: We have revised the statement in the submission system according to the journal requirements found in the referenced page link. Please let us know if something is still missing and we can update.

2. We noticed that you used "data not shown" in the manuscript. We do not allow these references, as the PLOS data access policy requires that all data be either published with the manuscript or made available in a publicly accessible database. Please amend the supplementary material to include the referenced data or remove the references.

**Response**: We have removed reference to “data not shown” on page 28.

**Reviewer comments**

**REVIEWER #3:** big problem of the manuscript is a statistical bias. As said in the previous revision, the data need to be weighted seriously.
Sampling
Done For autoweighted Weight
Pub Privé Tot Pub Privé Tot Pub Privé
Centre 8 8 3 16 0,423 2,061
Hauts-Bassins 8 8 5 7 0,593 0,923
Total 32 32

Regarding the weight, it is not possible to have the same results in present manuscript. This embarrassed me. Purhaps, it will be better to have advise with an other reviewer expert in quantitative data.

Statistical bias affects the whole result. As the sample was done, without weighting it is not appropriated to conclude for the whole area.

**RESPONSE**: From your comments, we believe that you are concerned that the schools we recruited into our study sample are not representative of schools in Bobo and Ouaga. We did several things to ensure a representative sample and ensure school type was considered, including a thorough mapping exercise to develop our sampling frame of private and public schools in our project catchment areas; including school type as a key variable in our analysis and using effect modification to understand how school type may differentially impact girls.

We followed the standard procedures to undertake individual-level analysis of a multi-stage cluster design, using schools as clusters, as described by Hayes and Moulton (2017). As we describe in our methods section, in the first stage we randomly selected health facilities from a list of purposively sampled health facilities in our project catchment area, based on facility type and geographic spread, including facilities offering youth-friendly services. To reach our sample size, we then selected from 32 schools (16 in each city) – half were randomly assigned to be intervention schools, and the other half were comparison schools. We randomly assigned a higher proportion of private schools, as fewer public schools in Bobo met our criteria. We used power calculations to identify the number of individuals within schools (clusters). Our primary concern in a CRT is to ensure we have enough clusters; similar, more or less, numbers of individuals within clusters; and that control and intervention clusters do not differ in some key characteristics at baseline.

Given that the unit of intervention in a CRT is the cluster, a weighted analysis using this study design could be considered if the analysis was based on cluster-level summaries with a considerable unequal number of participants in each cluster. Therefore, we do not feel it is appropriate to weight this analysis because (a) our analysis was based on individual-level data using GEE that takes into account correlations between observations in the same cluster, (b) we had enough clusters per intervention with approximately equal sample sizes to ensure that individual-level analysis is robust and more accurate than a cluster-level analysis, (c) intervention and control clusters (ie. unit of intervention) were well balanced in key characteristics and (d) given that we achieved to have an approximate equal number of participants between clusters, a weighted approach would reduce accuracy and increase sampling variance, standard deviation and standard error.

However, we understand that we may not fully have addressed the potential selection bias. In a previous round of review, we updated the methods section to include more information up front about out process. After this review, we have added the following to the limitations section:

Page 29. “Several factors should be considered in understanding the results of this evaluation. Generalizability of results may be limited to some in-school girls in larger cities in West Africa. First, although we completed a mapping of schools before data collection to inform our sampling frame, it is possible our final sample does not reflect the true distribution of public/private schools in both cities. This is partly due to selecting schools within the catchment areas of our project health facilities. In addition, the effect modification analysis suggests differential impact by school type; therefore, caution should be taken when generalizing to all schoolgirls in public and private schools.”

Reference: Hayes RJ, Moulton, LH. Cluster Randomised Trials, Second Edition. CRC Press. 2017. Available from: <https://doi.org/10.4324/9781315370286>
