# Supplementary material for: Does a school-based intervention increase girls’ sexual and reproductive health attitudes and intentions? Results from a mixed-methods cluster-randomized trial in Burkina Faso
Source: PLOS Glob Public Health. 2023 Dec 11;3(12):e0000910. doi: 10.1371/journal.pgph.0000910 (PMC10712850; doi:10.1371/journal.pgph.0000910)
Supplement: Attachment — Submitted filename: Response to reviewer Jan 30 2023.docx [file pgph.0000910.s005.docx]

Reviewer #3: To your response: I'm not convinced with your answer.
It is known that pupil who attend private schools are more some of who failed in public schools. Because of that there are more aged than others. They are aware of sex experience and have some knowledge on reproductive health. They are more well informed on SRH. This implies that girls from private schools have more attention on SRH. Some schools have more aged girls in grades 4e and 3e. The sample frame is not well described because girls selection in school and grade is not known. Schools size varied, how girls are selected ? This is a lack for the methodology

We understand your concerns about the differences between private and public schools and the fact that pupils in private schools are more likely to be older and probably more likely to be informed about SRH, which could confound the relationship between the intervention and our outcomes.

We have addressed your concerns in two ways.

First, we addressed this potential bias of age and grade at the analysis stage where we developed models that are adjusted for age (Model 1) and additionally adjust for grade (model 3). This approach ensures that any confounding effect of age (and grade) on our outcomes is taken into account. Furthermore, we performed stratified analysis by type of school (private vs public) to identify if the impact of the intervention on our outcomes was different between public and private schools. This is in line with your concerns about differential impacts between the two types of schools, ie. our analysis examines whether there are and to what extent differences in our outcomes between public and private schools.

Please see the second paragraph in the statistical analysis section. Specifically, we state “*For the main analyses, we examined the association between key sociodemographic and attitudinal predictors and the primary outcome within the regression framework described above. We used generalized estimating equations (GEE) analysis to examine the impact of the intervention on our primary outcome of interest. We developed four models, all of them adjusted for baseline values of the outcome. In the first model (unadjusted model) we report the crude estimate of the impact of the intervention on our outcome of interest.* ***For the adjusted analyses, we first fit models adjusted for age (adjusted Model 1).*** *We then additionally adjusted for predictors that were found to have a statistically significant association with our primary outcome (e.g. COVID-19’s effect on mobility, adjusted Model 2).* ***In the final model we additionally adjusted for predictors that were selected a priori based on theory and previous research (e.g. city, grade, and wealth quintile, adjusted Model 3).*** *We fit all models with exchangeable correlation matrix (to allow for correlations between participants within the same cluster) and robust standard errors, as recommended for cluster randomized trials with more than 15 clusters per arm [18].*

*In* ***sensitivity analyses****, we ran our* ***models stratified*** ***by several variables for which we thought the impact might vary by, including*** *reporting ever having had sex,* ***type of school (i.e. private or public),*** *grade and whether girls in the intervention schools visited a health facility.*” We have now also added the sentence “*These models were adjusted for* ***age****, city, COVID-19’s effect on mobility and wealth quintile.* “. Please also see the last paragraph in the results in the main analysis section where we report that “*we found the outcome differs significantly between girls in control and intervention schools* ***depending on school type****. The impact of the intervention was greater in the private schools (i.e. girls in intervention schools were more likely to report intention to use contraception in the next three months compared to girls in the control schools) compared to the public schools (i.e. no difference between control and intervention schools) (P<=0.001).*”.

Second, we acknowledge in the discussion that the selection of schools and pupils may have led to bias. In the third paragraph of the discussion, we have added the following sentence “*Given what is known about private schools in urban areas in Burkina Faso, it could be that girls in private schools are older or have more maturity and experience related to sexual and reproductive health*;”. In the section “Considerations for the Evaluation” we also report that “*First, although we completed a mapping of schools before data collection to inform our sampling frame, it is possible our final sample does not reflect the true distribution of public/private schools in both cities. This is partly due to selecting schools within the catchment areas of our project health facilities.*”

In terms of the sampling frame, we have a detailed section on “Selection of (re)solve health facilities, schools, and participants” where the sampling frame and data collection is described. In terms of how girls are selected, we state, “*Once the full list of interested girls was prepared, the team randomly sorted the list and then invited the first set of girls on the list to participate in the quantitative survey, followed by the qualitative study. At control schools, all girls in relevant grades interested in participating in the research were put on a list and that list was randomly sorted, and the girls whose names were at the top of the list were invited to participate.*”. We also performed power calculations to select the number of schools (clusters) and the number of participants per school. These are all described in detail in the methods section.
