# Supplementary material for: Does a school-based intervention increase girls’ sexual and reproductive health attitudes and intentions? Results from a mixed-methods cluster-randomized trial in Burkina Faso
Source: PLOS Glob Public Health. 2023 Dec 11;3(12):e0000910. doi: 10.1371/journal.pgph.0000910 (PMC10712850; doi:10.1371/journal.pgph.0000910)
Supplement: Attachment — Submitted filename: Response to Reviewers.docx [file pgph.0000910.s006.docx]

Dear Jianhong Zhou
Staff Editor
PLOS Global Public Health,

Thank you for the opportunity review and revise our manuscript. We have addressed reviewers’ comments below on a point-by-point basis. In addition, we have taken this opportunity to update other aspects of the paper including a few author affiliations. These changes are noted in track changes and in the clean version of the manuscript. Please do not hesitate if you have any questions.

With appreciation,

Laura Hinson and the authorship team

Comments to the Author:

Reviewer #4: Abstract:
• Line 13: missing word: Compared (to) girls…

**RESPONSE: We have added the missing word.**

Introduction:
• Sentence structure: Girls or women age(d) 15-19 or 15-24.
• Page 7, line 5: four years after having sex for the first time (median age 18.1), and over a year after getting married (median age 20.9).

**RESPONSE: We have updated our language.**

• Page 7, Line 13: suggested edit: Nearly all studies included in the review were HIV-focused, and there were almost no school-based interventions focusing on contraception and risk perception around pregnancy for girls from Burkina Faso or the West Africa region in general.

**RESPONSE: We agree that the literature is scant on school-based SRH interventions in SSA, especially evidence from West Africa. We had tried to express this in the paragraph but see through new eyes how we did not succeed in making the point well. We have gone back to the literature, updated this section (removing a few references we felt did not serve the paragraph well, and adding in a few others), and rewrote the text. We hope our message comes across better this time!**

• I think that the introduction is a more suitable place to discuss the (re)solve program than the methods section. Please provide some information about the program components as well as its theoretical underpinning. If there are previous studies discussing the design of the program, refer the reader to them in the introduction. There is no need to provide a lot of detail in that case.

**RESPONSE: We have relocated the sub-section covering the (re)solve intervention from the Methods sections to the Introduction. This sub-section provides an overview of the components of the intervention, including more details on how it was developed. We have added bullet points for each component to this section to help delineate them.**

• While intention is a good predictor of contraceptive use among girls and women, it would be great if the references were specific to the Sub-Saharan Africa region, as region-specific factors may be at play regarding the translation of intention into action (e.g.: social support, perceived norms). Additionally, none of the citations are specific to adolescent girls, who are the target of the intervention.

**RESPONSE: Please note that one of the references already in this section *(Ross JA, Winfrey WL. Contraceptive use, intention to use, and unmet need during the extended postpartum period. Int Perspect Sex Reprod Health. 2001;27(1):20-27.)* is based on secondary analysis of Demographic and Health Surveys (DHS) from 27 Low- and Middle-Income Countries (LMICs), including 12 countries from the Sub-Saharan Africa region. This article states that a positive relationship exits between intention to use and contraceptive use. To further strengthen our statement that ‘intention to use contraception… is often predictive of future use” (pg8), we have added a new reference (Negash, W.D., Eshetu, H.B. & Asmamaw, D.B. Intention to use contraceptives and its correlates among reproductive age women in selected high fertility sub-saharan Africa countries: a multilevel mixed effects analysis. BMC Public Health 23, 257 (2023)) which looks at the topic of intention to use specific to the Sub-Sahara Africa region and makes an important note that age is a correlate with intention to use.**

Methods:
• Expand the section on the qualitative interviews and explain the kinds of questions each group of participants was asked and where these questions came from, as well as how participants were selected to take part in the interviews.

**RESPONSE: We have expanded the qualitative methods section (page 14) to include the types of questions asked of each group. All selection information for qualitative participants is noted on page 14.**

• Is there a reason why interviewers were mixed-gendered? I would assume that girls may be too shy to discuss questions of sensitive nature with male interviewers. Was there any analysis to determine whether the interviewer’s gender impacted the responses in any way?

**RESPONSE: This is an important point, and one that we are often concerned about when conducting SRH research. Our data collection team assured us that this would not be an issue. We piloted the interviews using the mixed-gendered team, and in addition, we quantitatively assessed the first tranche of data to see if there were differences by gender, and we found none. We have added in a note to this effect in the manuscript.**

Results:
• Page 21: table 3: I feel confident in my ability to use and get a contraceptive method, if I wanted to (not) get pregnant.

**RESPONSE: Good catch, we have updated this error.**

• Table 3: the text of table 3 needs to include more information. Are there any statistically significant differences between control and intervention groups on any of the variables?

**RESPONSE: Thank you for your comment. In Table 3 we present trends between baseline and endline. We now report in the methods that “***We examined differences in community-level geometric means between control and intervention schools separately at baseline and endline using the Wilcoxon rank-sum test with 1000 Monte Carlo permutations.*” **We used Wilcoxon rank-sum test with 1000 Monte Carlo permutations because we are reporting the geometric means. We have also added footnotes under Table 3 to highlight statistically significant differences between control and intervention schools in the list of variables at baseline and endline.**

• I feel that the qualitative section needs a more in-depth analysis. It would be good to highlight the themes that were common/different across different groups of participants (e.g., girls in public versus girls in private schools, sexually active versus non-sexually active girls) and explain whether the findings are consistent with the quantitative results. Second, there was barely any mention of the themes that emerged from the interviews with key informants or with implementation staff. Overall, the value of the qualitative interviews and how they complement and support the quantitative findings needs to be further emphasized.

**RESPONSE: Due to the small sample size of the qualitative participants, it was not feasible to subdivide the group by demographic characteristics - for instance, the number of sexually active girls was very small. We have added language in several places noting where the qualitative and quantitative data align. The primary data for this analysis is the quant data, with qual used to supplement – given space, we were not able to fold in much qualitative and feel that is best served in a stand-alone paper. Relatedly, KII and implementer data are less relevant as those interviewers were focused on implementation more than impact. The points are well taken though, and so we have added in some description of our analytic approach to this effect, which we hope will clarify how we use the methods and data in this paper (page 15).**

Discussion:
• “Given what is known about private schools in urban areas in Burkina Faso, it could be that girls in private schools are older or have more maturity and experience related to sexual and reproductive health”. This point needs more elaboration and references if available. Not everyone is familiar with Burkina Faso’s private schools demographics. Why would private school girls be older or more mature than public school girls? Do girls enroll in private schools when they are performing poorly and repeating 4eme and 3eme grades? Are girls in private schools coming from wealthier families? Are their parents more likely to have a higher level of education than those of girls who are enrolled in public schools? What are some possible explanations for this observation?

**RESPONSE: We have conducted a literature review to see if there are any articles, either in peer-reviewed journals or in the gray literature, to shed some insights into the difference between private and public schools and potential patterns with sociodemographic and reproductive health characteristics of students. Unfortunately, the intersectionality between student socio-demographics, school quality, and reproductive health behaviors are not well documented or understood. As a result, we feel that it would be inappropriate to provide possible explanations for these observations without concrete references. We have added the following sentence to highlight the need for future research in this important area at the end of this paragraph: “Understanding the intersectionality of school type, sociodemographic characteristics of students, and patterns of reproductive health behaviors in Burkina Faso, or within the sub-Saharan Africa region, is an important topic of future research.” (pg 28)**

• “Even though our previous behavioral diagnosis data indicated that girls would be sexually active, we did not find this to be true in our evaluation data.” What may be the reason for that? Are there any substantial differences between the girls from your diagnosis data and those participating in the evaluation?

**RESPONSE: This is a good question, and one that we spent quite a bit of time trying to understand. The girls in the diagnosis sample and the ones in the evaluation sample were similar, although from different schools. The diagnosis sample was mostly qualitative in nature and the research team had more time to build rapport and have an open-ended, flowing discussion. As being administered a quantitative survey can be less personal, we are not surprised to find that fewer may have felt comfortable reporting sexual activity. Our qualitative data in the evaluation study also showed higher levels of reporting of sexual activity, which confirms our hypothesis. We have added a note to this effect in the manuscript.**
