# Supplementary material for: Does a school-based intervention increase girls’ sexual and reproductive health attitudes and intentions? Results from a mixed-methods cluster-randomized trial in Burkina Faso
Source: PLOS Glob Public Health. 2023 Dec 11;3(12):e0000910. doi: 10.1371/journal.pgph.0000910 (PMC10712850; doi:10.1371/journal.pgph.0000910)
Supplement: Attachment — Submitted filename: Reviewer comments (7.14.22).docx [file pgph.0000910.s003.docx]

Journal Requirements:

1. Please include a complete copy of PLOS’ questionnaire on inclusiveness in global research in your revised manuscript. Our policy for research in this area aims to improve transparency in the reporting of research performed outside of researchers’ own country or community. The policy applies to researchers who have traveled to a different country to conduct research, research with Indigenous populations or their lands, and research on cultural artifacts. The questionnaire can also be requested at the journal’s discretion for any other submissions, even if these conditions are not met.  Please find more information on the policy and a link to download a blank copy of the questionnaire here: [https://journals.plos.org/globalpublichealth/s/best-practices-in-research-reporting](https://nam10.safelinks.protection.outlook.com/?url=https%3A%2F%2Fjournals.plos.org%2Fglobalpublichealth%2Fs%2Fbest-practices-in-research-reporting&data=05%7C01%7CBBrooks%40pathfinder.org%7Cb408076beb184483edb508da2f60f0d1%7Cf3f7b6d34d5e46c6826c78d1a7e3bc6a%7C1%7C0%7C637874391222573425%7CUnknown%7CTWFpbGZsb3d8eyJWIjoiMC4wLjAwMDAiLCJQIjoiV2luMzIiLCJBTiI6Ik1haWwiLCJXVCI6Mn0%3D%7C2000%7C%7C%7C&sdata=MeHcAeJUWjIuexlDBtVqNmPBN0UoC%2Fvj7SNyOGdFpdw%3D&reserved=0). Please upload a completed version of your questionnaire as Supporting Information when you resubmit your manuscript

**RESPONSE:** We have completed and uploaded the questionnaire on inclusiveness.

2. We recommend authors use the COREQ checklist, or other relevant checklists listed by the Equator Network, such as SRQR, to ensure complete reporting. For additional information, please check [http://journals.plos.org/globalpublichealth/s/submission-guidelines#loc-qualitative-research](https://nam10.safelinks.protection.outlook.com/?url=http%3A%2F%2Fjournals.plos.org%2Fglobalpublichealth%2Fs%2Fsubmission-guidelines%23loc-qualitative-research&data=05%7C01%7CBBrooks%40pathfinder.org%7Cb408076beb184483edb508da2f60f0d1%7Cf3f7b6d34d5e46c6826c78d1a7e3bc6a%7C1%7C0%7C637874391222573425%7CUnknown%7CTWFpbGZsb3d8eyJWIjoiMC4wLjAwMDAiLCJQIjoiV2luMzIiLCJBTiI6Ik1haWwiLCJXVCI6Mn0%3D%7C2000%7C%7C%7C&sdata=1d7eHlcQVgUXWY2Wb20O4MJnsr1pLMZ9b6EzOq5YQsI%3D&reserved=0)

**RESPONSE:** We used a modified version of the COREQ checklist for reporting methods to add additional information to the methods section. Given the length of the paper and considering it is a mixed methods study, we have included some but not all information pertaining to the 32-item checklist. We selected the items we felt were of most important to PLos One readers. If there is additional information the editorial team would like to see, we can add it into the manuscript.

3. Please update your Competing Interests statement. If you have no competing interests to declare, please state: “The authors have declared that no competing interests exist.”

**RESPONSE:** We have updated our declaration.

4. In the online submission form, you indicated that “The datasets used and/or analyzed during the current study are available from the corresponding author on reasonable request.”. All PLOS journals now require all data underlying the findings described in their manuscript to be freely available to other researchers, either 1. In a public repository, 2. Within the manuscript itself, or 3. Uploaded as supplementary information.
This policy applies to all data except where public deposition would breach compliance with the protocol approved by your research ethics board. If your data cannot be made publicly available for ethical or legal reasons (e.g., public availability would compromise patient privacy), please explain your reasons by return email and your exemption request will be escalated to the editor for approval. Your exemption request will be handled independently and will not hold up the peer review process, but will need to be resolved should your manuscript be accepted for publication. One of the Editorial team will then be in touch if there are any issues.

**RESPONSE:** We will make the data available and will upload as supplementary information. We need a bit of time to prepare the data but should the article be accepted we will ensure the data is ready for public availability before publication.

5. We noticed that you used “data not shown” in the manuscript. We do not allow these references, as the PLOS data access policy requires that all data be either published with the manuscript or made available in a publicly accessible database. Please amend the supplementary material to include the referenced data or remove the references.

**RESPONSE:** We have deleted reference to “Data not shown.” We meant to infer that there is no corresponding table with the data but the relevant information we want to convey is there.

6. Please provide separate figure files in .tif or .eps format only and ensure that all files are under our size limit of 10MB.

**RESPONSE:** We have done as requested.

For more information about how to convert your figure files please see our guidelines: [https://journals.plos.org/globalpublichealth/s/figures](https://nam10.safelinks.protection.outlook.com/?url=https%3A%2F%2Fjournals.plos.org%2Fglobalpublichealth%2Fs%2Ffigures&data=05%7C01%7CBBrooks%40pathfinder.org%7Cb408076beb184483edb508da2f60f0d1%7Cf3f7b6d34d5e46c6826c78d1a7e3bc6a%7C1%7C0%7C637874391222573425%7CUnknown%7CTWFpbGZsb3d8eyJWIjoiMC4wLjAwMDAiLCJQIjoiV2luMzIiLCJBTiI6Ik1haWwiLCJXVCI6Mn0%3D%7C2000%7C%7C%7C&sdata=%2BXvVTKnRvbxxyYlCqDalgag0au%2BZ5q%2BB6MFywVi6s%2BM%3D&reserved=0)

5. Review Comments to the Author

Please use the space provided to explain your answers to the questions above. You may also include additional comments for the author, including concerns about dual publication, research ethics, or publication ethics. (Please upload your review as an attachment if it exceeds 20,000 characters)

**Reviewer #1:**

1. Summary of the Research and Overall Impression
This is an evaluation of the sexual and Reproductive Health interventions for the girls in grades 9 and 10 in private and public schools in Burkina Faso. It is stated that there was positive reception of the interventions such that at the end of the intervention implementation, girls aged 14-18 years in the intervention group had positive attitudes towards modern contraceptives. The impact of the intervention is more prominent among the girls who have never had sex which makes it easy because by the time they engage into sexual relationships, they will be fully aware of methods of contraceptives and be free to attend the health services (adolescent friendly).
Methodology including sample size is well described. The results section shows that majority of girls received almost all the interventions and were able to contribute in distributing the health passport for other girls to benefit from the intervention. It is evident that most attitudes towards contraceptive use were significantly associated with the intension to use in the next three months. This was almost the same among girls who have visited health facilities and those who did not visit them due to various reasons.
This is good evaluation that is going to generate evidence on the SRH knowledge, attitudes and behaviors especially in the West Africa. I find the evaluation to helpful to Burkina Faso in that the positive reception of the SRH interventions will play a role in reduction of high teenage pregnancy and other SRH issues such as sexual transmitted diseases. This evaluation will also influence adoption of adolescent friendly health services country wide. The girls who were part of the intervention will also spread the work to other girls and eventually majority will be transformed and consequently contributing to the reduction of high teenage pregnancy.
The interventions are well articulated such that it will be easy to replicate or roll out countrywide.

**RESPONSE:** Thank you for your supportive comment!

2. Evidence and Examples
a. Major Issues
i. No Major issues
b. Minor Issues
i. Full title: The authors need to be specific on what intervention are they talking about that increases girls’ sexual and reproductive health attitudes and intentions

**RESPONSE:** We believe the current title accurately describes the “school-based” intervention: “Does a school-based intervention increase girls’ sexual and reproductive health attitudes and intentions? Results from a mixed-methods cluster-randomized trial in Burkina Faso”. However, we did make minor changes in the description of the intervention to highlight the point that this is indeed a “school-based” intervention:

“The (re)solve intervention was comprised the following components: a **school-based** participatory board game (La Chance) that corrected SRH myths and misconceptions and increased pregnancy risk perception; a health passport **given at schools** that eased girls’ access to health facilities; posters in schools and health facilities that advertised consultations for adolescent girls to address menstruation and other puberty-related concerns; and name tags that identified youth-friendly health care providers.” (Pg 7)

ii. Line 173: The authors show that participants aged less than 20 they obtained parental consent. One would just wonder if the age of adults in Burkina Faso is 20 years and above

**RESPONSE:** We have made the following changes to clarify this sentence:

“Under the guidance of CEIRSS, we set the age of majority at 20 years. Therefore, all participants aged 20 and older signed a consent form; for girls younger than 20, we obtained parental consent and then girls’ assent for participation. During the assent and consent processes, participants and, when relevant, their parents were made aware that the researcher was part of the REM Africa team, working with ICRW, and conducting a study related to (re)solve.” (Page 10)

iii. Lines 177-183: endline data was collected in July 2020 due to covid-19 as it was supposed to have happened in March and I am just wondering if covid-19 could have had an impact on the results.

**RESPONSE:** This is an important comment. To assess the potential effect of COVID-19 on our primary outcome of interest (intention to use contraception), we collected data on how COVID-19 may affect mobility of our study participants. In addition, we made sure that our adjusted regression model accounted for “COVID-19 effect on mobility”. Our analyses suggest that COVID-19 has had limited effect on the primary outcome of interest. We also note in the discussion that, based on our data, we have reason to believe the pandemic was a reason why some girls did not follow up at a clinic for SRH services (page 29).

iv. Line 186: The authors purposely selected 9 health facilities, could there be justification of why purposive sampling, why not random sampling or any other sampling, maybe it can come out clearly in that section.

**RESPONSE:** We have added a new sentence to clarify the reason behind our purposive sampling approach:

“In the first stage, the team purposively selected nine health facilities in Ouaga and 10 in Bobo from a list of eligible facilities. **The purposive sampling was used to ensure that we had diversity of health facilities based on facility type and geographical spread, and to make sure that these facilities all offered youth friendly services**.” (Pg 8)

v. Line 294: they are mentioning Figure 1 and line 299 has an instruction to insert Figure 1, could it be the authors forgot to include the figure?

**RESPONSE:** Thank you for this comment. This is a copy-edit note to ensure the appropriate placement of Fig 1 (which was submitted in this manuscript application as a separate file). The final version of this manuscript will not have the “insert fig 1” note on this page. Fig 1 has now been inserted into the revised manuscript

vi. In the results section authors refer to Figures but I cannot see any mentioned Figures in the document. However, in the end there is a link provided for readers to download the figures and tables referred to in the text. I recommend the Figures to be part of the text as it becomes easier for a reader to refer to the figure when reading the text.

**RESPONSE:** Thank you for this comment. In order to improve the readability and flow of information, while balancing the availability of data and transparency of our analysis, we believe that it will be most useful to include the ten (10) supplemental figures and tables in the annex.

**Reviewer #2:**

General comments: This RCT is a well-presented example of rigorous, in-school interventions recommended by international frameworks such as UNESCO’s International Technical Guidance of sexuality education to provide evidence for designing school-based sexual and reproductive health (SRH) interventions. The mixed-methods approach sought to assess the behavior change effectiveness of a board game on adolescents’ intentions to use contraception, intention to engage with health service facilities on contraception, and other related sexual health issues such as puberty and menstruation, and sexual activity.

**RESPONSE:** Thank you for your supportive comment!

Please find below the following minor suggestions to increase strength in some areas of the paper:
1. Pg 7, Lines 146-147- The involvement of health care professionals to provide user-friendly services for adolescent participants of the intervention:
Your intervention sought to engage with health workers to assess young people’s visits to health facilities. However, does this approach not introduce any bias in the attitudes of health workers? Indeed, the evidence elsewhere has established the negative impact of health workers on young people’s utilization of sexual health services, but these health workers are aware of the possible intentions of young people to visit health care facilities, and therefore are inclined to exhibit positive attitudes. I suggest an acknowledgement of this bias as a limitation in the ‘consideration for evaluation’ (P 552 ff).

**RESPONSE:** Thank you for this important comment. Agreed, any intervention targeted to health care professionals such as youth friendly service (YFS) training would inevitably change their attitudes towards youth clients to exhibit more positive behavior. However, we don’t think this would bias or effect the evaluation results since our sampling frame was based on schools that were located close to health facilities that provided YFS training. It is for this reason that our sampling approach first started with health facilities before randomly selecting schools into the intervention and control arm. “In the first stage, the team purposively selected nine health facilities in Ouaga and 10 in Bobo from a list of eligible facilities. The purposive sampling was used to ensure that we had diversity of health facilities based on facility type and geographical spread, and to make sure that these facilities all offered youth friendly services” (Page 8). Since we were implementing a school-based intervention targeted to young schoolgirls that could result in increased number of SRH visits in surrounding health facilities, we believed from both a programmatic and ethical perspective (ie. “do no harm” principle to protect project participants) that it was important that all health facilities close to randomly selected control and intervention schools had providers trained in youth friendly services.

2. Pg 8. Lines 170-172- A point of note (not to be addressed by author): It is commendable that the authors sought ethics approval from an international review board and consulted with a local ethics board in the country (Burkina Faso), something lacking in some studies.

**RESPONSE:** Thank you for your supportive comment. We believe it is important for the ethical review boards in the countries where the research takes place to be involved.

3. Pg 9. Lines 190-192: This comment is on the selection of the majority of student-participants from private schools as compared to public schools. In some educational systems in West Africa, eg. Ghana and Nigeria, most State/Ministry of Education led sexual health programs are implemented in public basic schools, excluding private schools. If this situation pertains in the Burkinabe basic school system, it may affect some assumptions made at the baseline of the participants in the control groups and intervention groups, since there may be some variation in the student engagement in sexual health programs in the private and public schools. Please justify the selection of students in the private schools against this background, and also respond to this limitation (if applicable) in the 'consideration for evaluation'.

**RESPONSE:** This is an important comment. We included private schools in our study as our initial landscape analysis suggested that there was a greater proportion of secondary schools that were private in our urban setting of Ouagadougou and Bobo-Dioulasso. As a result, when we randomly selected secondary schools to be assigned into control and intervention arms, we had a higher proportion of private schools in our sample. We conducted exploratory analysis on our primary outcome of interest (intention to use contraception) between public and private schools and we noted an interesting observation: among private schools, the intervention was associated with increased intention to use while among public schools, the intervention was associated with decreased intention to use (Supplemental Table 3 – see below for quick reference).

Given what we knew about school types and grades in Burkina, we hypothesized that the outcome would be different by school type (i.e. private/public). We therefore explored this in sensitivity analyses (i.e. stratified) by treating school type as an effect modifier, which we describe in the method section: "We examined for interaction effects between intervention arm and the variables above, including key variables like attitudes, as we assumed that the impact of the intervention might be different based on responses to attitudes towards contraception use."

To be more forthcoming, we have added additional information about this aspect in the methods section and have included two plots looking at the interaction by arm of school and grade in the supplemental materials. We have also added one sentence in the discussion section to caveat implementation of the intervention in public versus private schooling (page 29).

4. P. 10. Lines 201-202:What is meant by this statement: ‘…the top girls on the list being invited to participate’.

**RESPONSE:** We have rewritten the sentence and hope it is clearer:

“At control schools, all girls in relevant grades interested in participating in the research were put on a list and that list was randomly sorted, **and the girls whose names were at the top of the list were invited to participate**.” (Page 8)

5. Pp 24 & 25. Lines 490-499. The authors present very interesting insights to suggest that while young people (in SSA and by implication Ghana and Burkina Faso) are known in the literature to be sexually active, there may be variations in sexual activity (ie. degrees of sexual intimacy). I think this is an important finding and should be emphasized in the discussion.

**RESPONSE:** We agree this is an important point, and we have added a statement in the discussion section to emphasis it more (page 27).

6. Pg 27 553-560. The authors discuss the extent to which the COVID 19 pandemic disrupted continuity in the intervention and may have resulted in the inability of girls to engage with health facilities. This finding presents an opportunity for the authors to extend the discussion to this conclusion-‘With the onset of the pandemic, there was a discontinuity/ a lack of focus in school-based SRH interventions and many young people did not have access to behavior and information change interventions. This may explain the high rates of adolescent pregnancies following the pandemic’.
This analogy is authentic, considering that the authors intend to generalize findings to West Africa, where the rise in adolescent pregnancies is very high, following the pandemic. This point can be drawn on in the discussions section, and you will be credited for providing a novel contribution to ongoing literature on the pandemic and its negative effects on inequities in health delivery.

**RESPONSE:** That the pandemic impacted our program and evaluation is an essential aspect to mention in the discussion. However, we are not able to extrapolate much given the timing of our research project and the outcomes we did (and did not) measure. It is likely that adolescent pregnancy increased after the pandemic but we don’t have the right kind of data to show this, unfortunately. We can only plant the seed of the idea, which we have done in the “considerations for the evaluation” section.

The authors are commended for the systematic and logical fashion in the discussion of the results.

**RESPONSE:** Thank you for your supportive comment!

**Reviewer #3:**

The sample is not clearly described because there is some missing. See suggestions for flowchart.
The real distribution of school children didn't meet the sample. This imply a weight calculation. This is not done.
Have a look on the distribution of school children by age for the difference with the sample. Adjustment is needed.
This implies some reserves for findings

**RESPONSE:** Thank you for noting some confusion on the flowchart, we have updated it and hope it is now clearer.

We are not sure we fully understand your comment about the weight calculation. This is a cluster-randomized trial with the unit of analysis being the cluster (school). In this type of study design, we are primarily interested in ensuring that the baseline control and intervention arms are well balanced in some key factors, which we present in tables and the results section. In our analysis, using the individual level data, we adjust for age in all models. We are unclear what other adjustments you might be referring to.

**Additional comments from a reviewer (taken from attached manuscript)**

Line 37: “implementation staff are not experts?”

**RESPONSE:** Thank you for pointing out this issue. Implementation staff are certainly experts, but we define experts in a different way for simplicity of reporting. More detailed information on participants is found starting (page 6). We have added more clarity on the distinction, which we clarify in a way that addresses your point. In the abstract we have character limits so will leave as is.

Line 40: “How about attrition because school people can communicated. Same family can have his children some in intervention and some in control. How can this be managed.”

**RESPONSE:** We are not sure we understand the first point. Your overall comment is related to the abstract – additional information about the results can be found in the full text. In terms of families with children in different schools, we assumed this scenario would be extremely unlikely so we did not choose to address it in our sampling and recruitment procedures. If there is evidence that the practice of sending children to different schools from the same family is common, we can note it as a limitation in our discussion section.

Line 46: “What this means? Naivety is not related to youngness”

**RESPONSE:** Sexually naïve means the person has little to no sexual experience. It is a descriptive term that can be applied to a person of any age. We feel the term is commonly enough used in the literature to remain as is.

Line 49: “If it is easy I think we only need to do project to change behavior”

**RESPONSE:** We are not sure we fully understand the comment. We do not mean to suggest our intervention is an easy fix for the challenge of adolescent pregnancy. Only that it may help to get young girls thinking about contraception at an early age.

Line 53: “Impact evaluation”

**RESPONSE:** Good suggestion, we have added this term to our key word list. Thank you!

Line 173: “Majority in Burkina Faso is 18. Why this choice? The consent of parent for 18-20 is nonsens”

**RESPONSE:** In consultation with CEIRSS, we were told to use the age of 20 as the age of consent. Although we agree in most places, the age of 18 is standard, we followed local guidance on this aspect. If there is indeed a discrepancy on the age of consent in Burkina Faso, we believe it is better to be more conservative than less in the case of consent of minors. We have updated the text around age of consent on page 10.

Line 192: “There is a bit sampling bias. Public is 17% in Ouaga (centre) and 39% in Bobo (Haut-Bassins). 25% is not good for the sample. School children in private are much elder than public. This could implie some choice”

**RESPONSE:** We agree there are differences between private and public schools in these two regions and this was considered in our analyses. We hypothesized that the outcome would be different by school type (i.e. private/public), grade and age. We therefore explored these in sensitivity analyses (i.e. stratified) as an effect modifier, which we describe in the method section: "We examined for interaction effects between intervention arm and the variables above, including key variables like attitudes, as we assumed that the impact of the intervention might be different based on responses to attitudes towards contraception use."

To be more forthcoming, we have added additional information about this aspect in the methods section and have included two plots looking at the interaction by arm of school and grade in the supplemental materials.

For sampling, we conducted a rigorous school mapping process that informed our decision of how many schools and of which type to include in our sampling frame. We adjusted for city in the final analyses.

We conducted a mapping exercise to inform or school selection approach. Based on the schools that were included in our sample, and given feasibility and logistics, we selected 25% public as our best percentage of what to include. We do adjust for city in the full analysis. We think this is sufficient for our study; however, we have added a sentence in the discussion section to address your point.

Line 197: “What this means? People can be interested because they are aware of that. This can imply the intention to change. Have a look at the distribution of people by age by class for public and private”

**RESPONSE:** We agree that a bias of our approach is that those girls who are interested to participate might already be more aware of or knowledgeable about sexual and reproductive health issues. However, we cannot avoid this bias because we cannot have girls participate who are not interested. In our assessment, this is so common of a bias that it does not merit mentioning in the limitations section.

We have assessed the sample of girls by age and class by public and private, see above.

Line 207: “This can yes if you have a look at their age. If most of them are youngest you can respond that to avoid critics”

**RESPONSE:** As it was only 12 girls, we did not feel it necessary to do further analyses on them. Instead, we went with the conversative approach and included them in the “no” category.

Related to Figure 1: “This need to be reviewed because it is not clear compared to what is described. The flowchart is: school, cities, type of school, level, trial, time”

Related to Figure 1: “Where is the 32 other? Why 32? Because 103-37 = 66.”

**RESPONSE:** Thank you for the careful review of our flowchart against the text. We agree that it is not entirely clear and have updated it accordingly.
